# ABCA1, ABCG1 and SR-BI: hormonal regulation in primary rat hepatocytes and human cell lines

**DOI:** 10.1186/1471-2199-8-5

**Published:** 2007-01-22

**Authors:** Marita Sporstøl, Seyed Ali Mousavi, Winnie Eskild, Norbert Roos, Trond Berg

**Affiliations:** 1 Programme for Cell Biology, Department of Molecular Biosciences, University of Oslo, Norway

## Abstract

**Background:**

Scavenger receptor type B class I (SR-BI), ABC transporter A1 (ABCA1) -and G1 (ABCG1) all play important roles in the reverse cholesterol transport. Reverse cholesterol transport is a mechanism whereby the body can eliminate excess cholesterol. Here, the regulation of SR-BI, ABCA1, and ABCG1 by dexamethasone (a synthetic glucocorticoid) and insulin were studied in order to gain more insight into the role of these two hormones in the cholesterol metabolism.

**Results:**

By use of real time RT-PCR and Western blotting we examined the expression of our target genes. The results show that SR-BI, ABCA1 and ABCG1 mRNA expression increased in response to dexamethasone while insulin treatment reduced the expression in primary rat hepatocytes. The stimulatory effect of dexamethasone was reduced by the addition of the anti-glucocorticoid mifepristone. In HepG2 cells and THP-1 macrophages, however, the effect of dexamethasone was absent or inhibitory with no significant change in the presence of mifepristone. The latter observation may be a result of the low protein expression of glucocorticoid receptor (GR) in these cell lines.

**Conclusion:**

Our results illustrates that insulin and glucocorticoids, two hormones crucial in the carbohydrate metabolism, also play an important role in the regulation of genes central in reverse cholesterol transport. We found a marked difference in mRNA expression between the primary cells and the two established cell lines when studying the effect of dexamethasone which may result from the varying expression levels of GR.

## Background

The process in which cholesterol is transported from peripheral cells, including those in the arterial wall, to the liver for excretion is termed reverse cholesterol transport. A key player in reverse cholesterol transport is the high density lipoprotein (HDL). The process can be divided into three stages: 1) the efflux of cellular cholesterol to HDL from peripheral cells, 2) the transport of HDL-cholesterol in blood to the liver, and 3) the delivery of cholesterol esters to hepatocytes from HDL [1]. The delivery of HDL cholesterol esters to the liver is mediated by Scavenger receptor class B type I (SR-BI). SR-BI also mediates selective uptake of HDL-cholesterol esters into adrenals, testis and ovaries [2,3] and cholesterol efflux to nascent HDL particles from macrophages and other peripheral cells [4,5]. SR-BI therefore plays an important role in both early and late stages in the reverse cholesterol transport pathway.

ATP-binding cassette (ABC) transporter A1 (ABCA1), abundantly expressed in the liver and peripheral macrophages among other tissues, mediates the transfer of cellular cholesterol and phospholipids to lipid-poor apolipoprotein A-I (apoA-I) to form nascent HDL particles [6-12]. ABCG1, another member of the ABC transporter family, has been suggested to be involved in cholesterol efflux to mature HDL and not to lipid-poor apoA-I [13,14].

Glucocorticoids are among the most widely used agents for the treatment of inflammatory and autoimmune diseases. Glucocorticoids exert their diverse effects through the glucocorticoid receptor, GR. GR belongs to the nuclear receptor superfamily and functions as a ligand-inducible transcription factor [15]. In the nucleus, activated GR can interact with the regulatory regions of responsive genes to alter the level of gene expression [16].

The aim of the present investigation was to study the role of the synthetic glucocorticoid dexamethasone in the regulation of ABCA1, ABCG1, and SR-BI. Here we show that dexamethasone-treatment increased the mRNA expression of ABCA1, ABCG1, and SR-BI in primary rat hepatocytes while an opposite tendency or no response was observed in HepG2 cells and THP-1 macrophages. We suggest that the observed inhibitory effect of dexamethasone is an unspecific/indirect effect since these cells express undetectable levels of GR protein. Moreover, the GR antagonist mifepristone had no significant antagonistic effect in these cell lines. The inhibitory effect of insulin on the mRNA expression was similar in primary rat hepatocytes, HepG2 cells and THP-1 macrophages.

## Results

Dexamethasone and insulin exert opposite effects on the mRNA expression of SR-BI, ABCA1, and ABCG1 in primary rat hepatocytes

**Figure 1:**
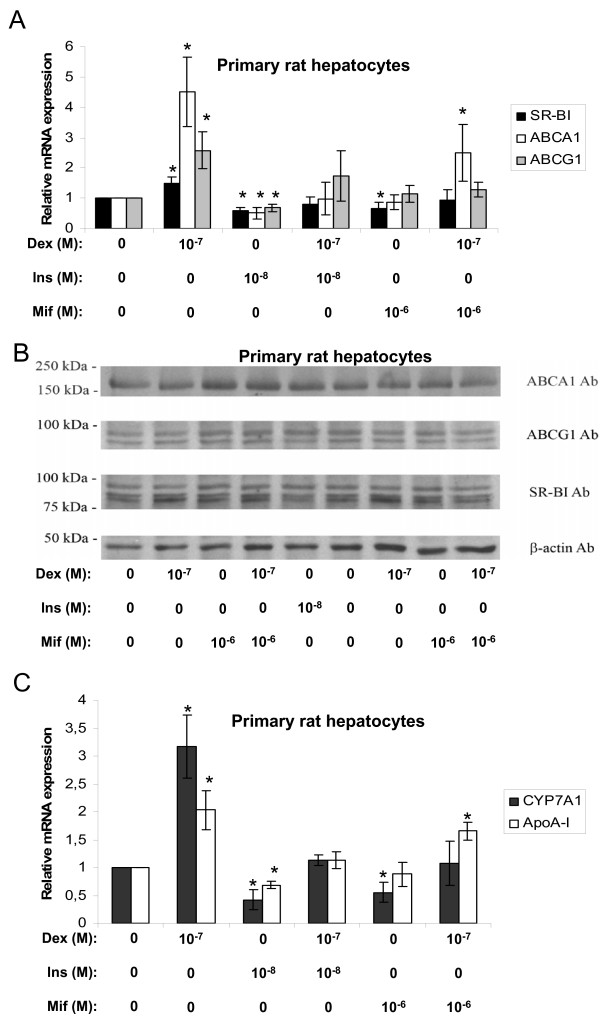
**Effects of dexamethasone, insulin or mifepristone in primary rat hepatocytes**. Primary rat hepatocytes were incubated with the indicated concentrations of dexamethasone (Dex), insulin (Ins), or mifepristone (Mif) in lipid-deficient medium for 24 hours and examined by real time RT-PCR and Western blotting. **A**. The relative mRNA expression of SR-BI (black bars), ABCA1 (white bars) and ABCG1 (gray bars). **B**. Representative Western blots of ABCA1, ABCG1, SR-BI and β-actin. Proteins (50 μg) were separated on a 7.5% polyacrylamide gel as described in materials and methods. **C**. The relative mRNA expression of CYP7A1 (black bars) and apoA-I (white bars). The results were normalized to GAPDH mRNA expression and expressed relative to the non-treated controls (medium B only) (± STDEV). The experiments were performed at least three times. * (p < 0.05) indicate significantly difference from control.

The expression of SR-BI, ABCA1, and ABCG1 were analyzed in primary rat hepatocytes by real time RT-PCR and Western blotting. As illustrated in figure 1A dexamethasone increased the abundance of SR-BI, ABCA1, and ABCG1 mRNA. Cells receiving insulin-treatment alone inhibited while treatment with both dexamethasone and insulin showed no significant effect on the mRNA expression levels of SR-BI, ABCA1 or ABCG1 compared to non-treated control cells indicating that insulin is capable of reversing the stimulatory effect of dexamethasone. In order to further evaluate the involvement of GR in the stimulatory actions of dexamethasone, mifepristone (RU38486), a progesterone and glucocorticoid receptor antagonist [17] was tested. The anti-glucocorticoid was shown to reduce the stimulatory effect of dexamethasone for all three target mRNAs (Fig. 1A). These treatments did not, however, notably alter the protein levels of SR-BI, ABCA1, and ABCG1 (Fig. 1B). Two well-documented HDL metabolism target genes for dexamethasone, CYP7A1 [18-21] and apoA-I [22-27], were also tested under the same conditions. As shown in figure 1C, the increased mRNA expression coincides well with previous reports demonstrating a stimulatory effect of dexamethasone on both CYP7A1 [18-21] and apoA-I [22-27] expression in rat liver. The addition of insulin to these cells reduced the mRNA levels and abolished the stimulatory effect of dexamethasone on both CYP7A1 and apoA-I expression. Again, the GR antagonist mifepristone reduced the stimulatory effect of the GR agonist dexamethasone. Thus the results presented in figure 1 show that dexamethasone and insulin have opposite effects on the mRNA expression of SR-BI, ABCA1, ABCG1, CYP7A1, and apoA-I in primary rat hepatocytes and that the stimulatory effect most likely is via binding and activation of GR.

In HepG2 cells and THP-1 macrophages dexamethasone and insulin have similar effects on the mRNA levels of SR-BI, ABCA1, and ABCG1

**Figure 2:**
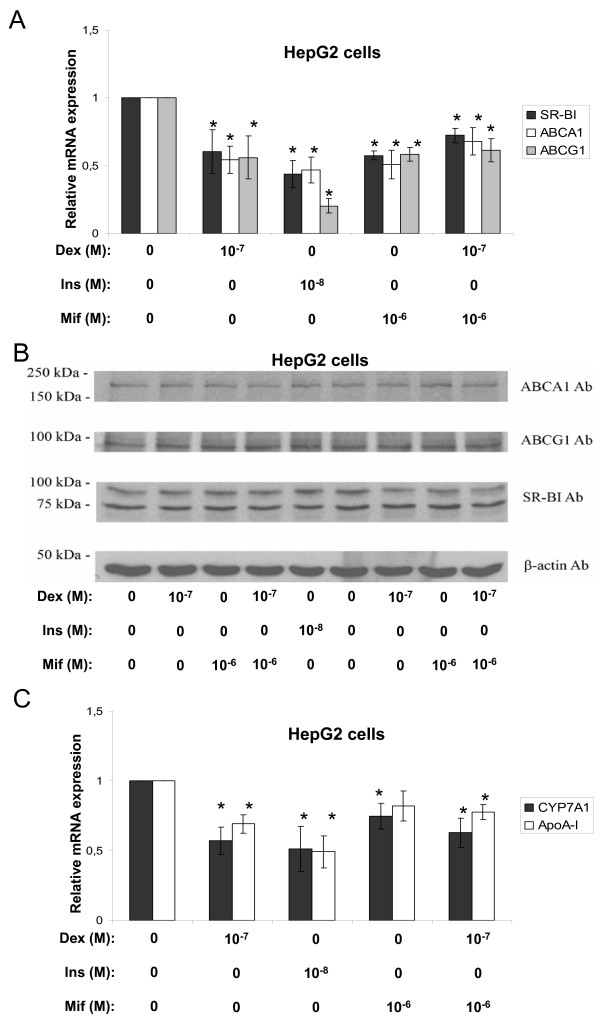
**Effects of dexamethasone, insulin, or mifepristone in HepG2 cells**. HepG2 cells were incubated with the indicated concentrations of dexamethasone (Dex), insulin (Ins), or mifepristone (Mif) in lipid-deficient medium for 24 hours and examined by real time RT-PCR and Western blotting. **A**. The relative mRNA expression of SR-BI (black bars), ABCA1 (white bars) and ABCG1 (gray bars). **B**. Representative Western blots of ABCA1, ABCG1, SR-BI and β-actin. Proteins (50 μg) were separated on a 7.5% polyacrylamide gel as described in materials and methods. **C**. The relative mRNA expression of CYP7A1 (black bars) and apoA-I (white bars). The results were normalized to GAPDH mRNA expression and expressed relative to the non-treated controls (medium B only) (± STDEV). The experiments were performed at least three times. * (p < 0.05) indicate significantly difference from control.

**Figure 3:**
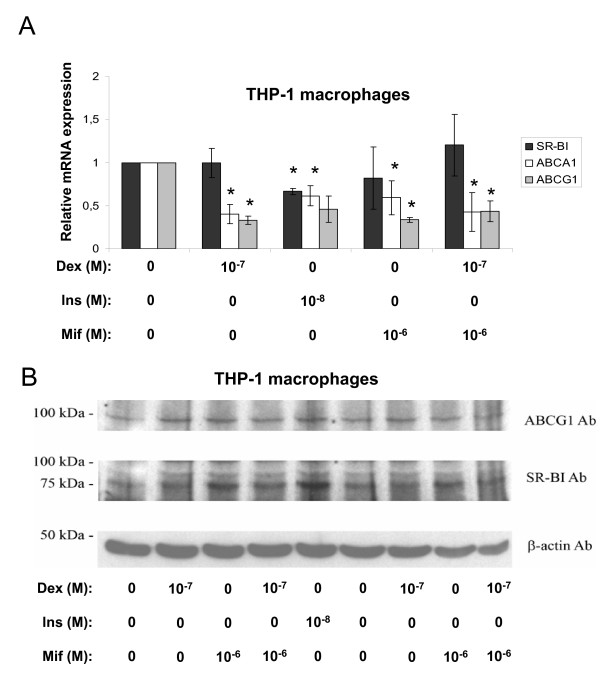
**Effects of dexamethasone, insulin, or mifepristone in THP-1 macrophages**. Macrophage-differentiated THP-1 cells were incubated with the indicated concentrations of dexamethasone (Dex), insulin (Ins), or mifepristone (Mif) in lipid-deficient medium for 24 hours and examined by real time RT-PCR and Western blotting. **A**. The relative mRNA expression of SR-BI (black bars), ABCA1 (white bars) and ABCG1 (gray bars). The results were normalized to GAPDH mRNA expression and expressed relative to the non-treated controls (medium B only) (± STDEV). **B**. Representative Western blots of ABCG1, SR-BI and β-actin. Proteins (50 μg) were separated on a 7.5% polyacrylamide gel as described in materials and methods. The experiments were performed at least three times. * (p < 0.05) indicate significantly difference from control.

**Figure 4:**
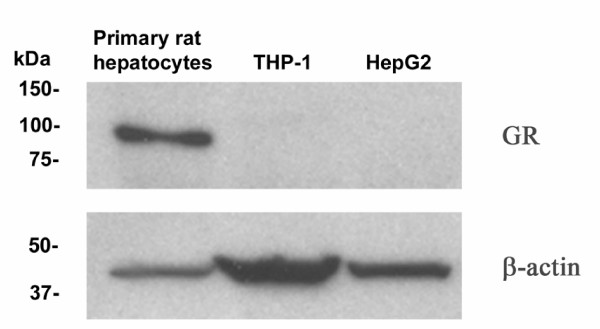
**GR protein expression**. Immunoreactive protein levels of GR and β-actin in primary rat hepatocytes, THP-1 macrophages and HepG2 cells were visualized by use of Western blotting. Proteins (25 μg) were separated on a 10% polyacrylamide gel as described in materials and methods.

Further observations in non-primary cells, such as the human hepatoma cell line HepG2 and the human macrophage-stimulated THP-1 cell line were carried out in order to test the effects of the two hormones in human-related cell systems. Surprisingly dexamethasone inhibited the mRNA expression of ABCA1 and ABCG1 in both HepG2 cells (Fig. 2A) and THP-1 macrophages (Fig. 3A). The SR-BI mRNA levels were reduced in HepG2 cells (Fig. 2A) while no effect could be observed in THP-1 macrophages (Fig. 3A). Again, no obvious change in protein levels could be observed for ABCA1, ABCG1 or SR-BI after these treatments for either HepG2 cells (Fig. 2B) or THP-1 macrophages (we did not observe any protein band for ABCA1) (Fig. 3B). The addition of mifepristone did not antagonize the dexamethasone effect for any of our target genes (Fig. 2A) or reference genes (Fig. 2C). Further experiments revealed, to our surprise, that the two cell lines indeed lack GR protein, at least compared to the expression level observed in primary rat hepatocytes (Fig. 4). The negative effects on mRNA expression observed with dexamethasone treatment in these cells (except for SR-BI mRNA in THP-1 macrophages) are therefore most likely unspecific or indirect. Thus, these findings link the stimulatory effect of dexamethasone to the endogenous expression levels of GR in primary rat hepatocytes. As observed in primary rat hepatocytes (Fig. 1), the effect of insulin was inhibitory on all the target mRNAs indicating that the insulin effect is intact in the established cell lines compared to the primary cells.

## Discussion

In this report, we have examined the regulation of a set of genes important in reverse cholesterol transport. Cells were treated with dexamethasone or insulin and mRNA and protein levels were quantified by use of real time RT-PCR and Western blotting, respectively. The data obtained show that dexamethasone treatment increased the mRNA expression of ABCA1, ABCG1, and SR-BI in primary rat hepatocytes while an opposite tendency was observed in HepG2 cells and THP-1 macrophages. It appears that the stimulatory effect of dexamethasone is specific/direct on the three target genes since it coincided well with the endogenous expression level of GR protein. This conclusion is further supported by the observation that the stimulatory effect of dexamethasone is reversed by the addition of the anti-glucocorticoid mifepristone. The negative effect of dexamethasone in THP-1 macrophages is in agreement with a previous study [28] indicating that dexamethasone alone reduced while co-transfection with GR alone increased the ABCA1 promoter activity in RAW264.7 and THP-1 macrophages.

By the use of classical Western blotting we were unable to detect any clear change in the protein expression of our target genes. As compared to real time RT-PCR, which can detect small changes in the levels of mRNAs, Western blotting is typically used for qualitative purposes and may therefore not display comparable changes in protein expression. The lack of effect of hormone treatments were nevertheless unexpected as earlier studies have shown that the protein levels of SR-BI respond within 24 hours to PPAR [29], FXR [30], and PXR [31] agonists. The dexamethasone treatment used in the present study should certainly be long enough to induce changes in protein levels. A plausible explanation for the lack of effect may be that it takes time before a detectable amount of protein is accumulated following dexamethasone treatment. This remains to be seen in future studies.

It is well recognized that glucocorticoids such as dexamethasone exerts catabolic actions while insulin exerts anabolic actions in carbohydrate metabolism. The present data show that dexamethasone and insulin may have opposite effects on cholesterol metabolism as well. One of the most important ways for the body to get rid of excess cholesterol is via the reverse cholesterol pathway and this pathway is tightly regulated by hormones and nutrients. Since SR-BI is the major route by which HDL-cholesterol esters can enter into the hepatocytes, and HDL-cholesterol is the preferred substrate for bile acid synthesis [32], the need for cooperative regulation of SR-BI and CYP7A1 is crucial in order to prevent accumulation of toxic high levels of bile acids. Our results suggest that dexamethasone increases reverse cholesterol transport by increasing genes central in these processes while insulin most likely has the opposite role by reducing the expression of the same set of genes. Moreover, our results are in agreement with a number of reports demonstrating that dexamethasone increases [18-21] while insulin decreases [19,20,33,34] the expression of CYP7A1, the rate-limiting enzyme in the conversion of cholesterol to bile acids, in rat liver cells. Also apoA-I expression increases in response to dexamethasone in primary rat hepatocytes as reported in numerous studies [22-27]. Our results show that insulin inhibits the mRNA expression of apoA-I in primary rat hepatocytes. This is in contrast to observations made in previous reports [22,23]. One study, however, shows that insulin at physiological concentrations and an incubation time of 12 hours inhibits the secretion of apoA-I from rat hepatocytes [24]. ApoA-I regulation by insulin may be affected by dose and time since Masumoto et al [24] and our results show that 10 nM insulin had an inhibitory effect after 12 and 24 hours, respectively. It appears that increased incubation time (44 or 96 hours) with both physiological and supraphysiological doses [22,23] switches the effect to be positive suggesting that insulin may have both fast and long term effects on target genes.

In summary, the present results suggest that dexamethasone and insulin have opposite roles in the regulation of genes participating in the reverse cholesterol pathway. It has been demonstrated that dexamethasone increases the internalization of HDL particles in rat hepatocytes [35] which may result from the increased hepatic expression of SR-BI observed here. Furthermore, the conversion of the accumulated cholesterol to bile acids is stimulated by the increased expression and activity of CYP7A1 [18-21]. This alone would be expected to result in reduced plasma HDL levels, but since the synthesis of apoA-I [22-27] and the mRNA expression of ABCA1 and ABCG1 is increased at the same time, it is not unreasonable to assume that the levels of plasma HDL actually increases. Thus dexamethasone-treatment, at least in short-time studies (24 h), may increase reverse cholesterol transport to get rid of the excess cholesterol and thus result in a healthier lipid profile with increased levels of plasma HDL.

## Conclusion

Our work demonstrates that the synthetic glucocorticoid dexamethasone increases the mRNA levels while insulin reduces the mRNA expression of SR-BI, ABCA1, and ABCG1 in primary rat hepatocytes. In the two human cell lines HepG2 and THP-1, however, the effect of dexamethasone was absent or inhibitory, illustrating an important difference in the use of cell lines vs. primary cells in the study of glucocorticoids. We suggest that this difference is a result of the endogenous expression levels of GR as illustrated by Western blotting and the use of the anti-glucocorticoid mifepristone.

## Methods

### Isolation and culturing of primary rat hepatocytes

Rats used in the experiments were treated according to established Guidelines for the Use of Experimental Animals. Primary rat hepatocytes were isolated from adult Wistar rats as described elsewhere [36]. After isolation, cells were grown in Dulbecco's modified Eagle's medium (DMEM) supplemented with 2 mM L-glutamine, 100 U/ml penicillin, 100 μg/ml streptomycin (Bio Whittaker, Europe), and 10% fetal bovine serum (Sigma) (medium A) at 37°C, 5% CO_2_. After 3–4 hours cells were stimulated with the given concentrations of dexamethasone, mifepristone (RU38486), or insulin (Sigma) in medium containing 0.5% charcoal-treated fetal bovine serum (medium B). Lipoprotein-depleted serum was prepared as described [37].

### Cell cultures

Human hepatoma cells, HepG2, were grown in DMEM supplemented with 2 mM L-glutamine, 100 U/ml penicillin, 100 μg/ml streptomycin, 1× non-essential amino acids (Bio Whittaker, Europe), and 5% fetal bovine serum (Sigma) (medium A) at 37°C, 5% CO_2_. After reaching 70–80% confluence, cells were stimulated with the given concentrations of dexamethasone, mifepristone (RU38486), or insulin (Sigma) in medium containing 0.5% charcoal-treated fetal bovine serum (medium B). The human monocyte-like cells (THP-1) were grown in RPMI-1640 supplemented with 2 mM L-glutamine, 100 U/ml penicillin, 100 μg/ml streptomycin (BIO WHITTAKER EUROPE) and 5% fetal bovine serum (Sigma) (medium A). The THP-1 cells were treated with 200 nM phorbol 12-myristate 13-acetate (PMA) (Sigma) for 72 hours to induce a macrophage phenotype. After reaching 70–80% confluence, the THP-1 macrophages were stimulated with the given concentrations of dexamethasone, mifepristone (RU38486), or insulin (Sigma) in lipid-depleted medium (containing 0.5% charcoal-treated fetal bovine serum) (medium B).

### Quantitative real time RT-PCR

Total RNA was isolated from primary rat hepatocytes, HepG2 cells or THP-1 macrophages using Versagene™ RNA Cell Kit (Gentra Systems) according to the manufacturer's instructions. Total RNA was reverse transcribed using SuperScript II RNase H^- ^Reverse Transcriptase (Invitrogen) and oligo dT primers (DNA Technology A/S, Denmark). The cDNA was used as template for quantitative real time RT-PCR on a Light Cycler (Roche) using SYBR Green I technology (Roche). The primers were designed by the Primer3 Program [38] and are listed in Table 1.

### Western blotting

Primary rat hepatocytes, THP-1 macrophages or HepG2 cells were washed twice with phosphate-buffered saline prior to lysis in lysis buffer (20 mM Hepes, 300 mM NaCl, 0.2 mM EDTA, 1.5 mM MgCl_2_, and 1% Triton X-100) containing 2% phenylmethylsulfonyl fluoride. Protein concentrations were determined by BCA protein assay (Pierce) and 25 or 50 μg of cell lysates were subjected to SDS-polyacrylamide gel electrophoresis on 7.5% or 10% polyacrylamide gels. Proteins were transferred onto nitrocellulose membranes (Millipore, USA) and pre-incubated with 5% skim milk phosphate-buffered saline-0.1% Tween 20 to prevent unspecific binding. Polyclonal rabbit anti-GR (P-20, Santa Cruz Biotechnology), anti-SR-BI (NB 400-104D, Novus Biologicals), anti-ABCA1 (400-105, Novus Biologicals), anti-ABCG1 (36969-100, Abcam), or polyclonal mouse anti-β-actin (AC-74, Sigma) were diluted in 5% skim milk phosphate-buffered saline-0.1% Tween 20 and interacting anti-rabbit or anti-mouse HRP-IgG (Amersham Biosciences) was detected by chemiluminescence ECL blot detection system (Amersham Biosciences). The blots were stripped with the Restore Western Blot Stripping Buffer (PIERCE).

### Statistics

Statistical significance of the data was evaluated by Student's t test. Probability values p < 0.05 was considered statistically significant.

## Authors' contributions

MS carried out most of the experiments and drafted the manuscript. SAM isolated and cultured rat hepatocytes and performed Western blots. WE participated in experimental design and data analysis. NR and TB supervised the study design and contributed to writing the manuscript.

## Figures and Tables

**Table 1:** Primers for quantitative real time RT-PCR analysis

**Gene**	**GeneBank accession**	**Forward primer (5'-3')**	**Reverse primer (5'-3')**	**Size (bp)**
ABCA1*		AACAGTTTGTGGCCCTTTTG	AGTTCCAGGCTGGGGTACTT	157
*h*ABCG1	NM_207630	GGTTCTTCGTCAGCTTCGAC	GTTTCCTGGCATTCAGGTGT	317
*r*ABCG1	NM_053502	GAAGGTTGCCACAGCTTCTC	CATGGTCTTGGCCAGGTAGT	339
*h*apoA-I	NM_000039	TGGATGTGCTCAAAGACAGC	AGGCCCTCTGTCTCCTTTTC	190
*r*apoA-I	NM_012738	CCTGGATGAATTCCAGGAGA	TCGCTGTAGAGCCCAAACTT	192
*h*CYP7A1	NM_000780	CCTCACCACACAGTCCCTTT	CCACACCTGGCTGTAATGTG	299
*r*CYP7A1	NM_012942	CGCTATTCTCTGGGCATCTC	GTACCGGCAGGTCATTCAGT	197
*h*GADPH	AF261085	GGCCTCCAAGGAGTAAGACC	AGGGGTCTACATGGCAACTG	147
*m*GADPH	BC023196	ACCCAGAAGACTGTGGATGG	CACATTGGGGGTAGGAACAC	171
*h*SR-BI	BC022087	CTGTGGGTGAGATCATGTGG	GCCAGAAGTCAACCTTGCTC	216
*r*SR-BI	NM_031541	CAAGAAGCCAAGCTGTAGGG	CCCAACAGGCTCTACTCAGC	230

* Primer sequences based on an alignment of human (*h*) (AF285167) and rat (*r*) (NM_178095) ABCA1.
